# Comparison of Fluorescent Microspheres and Colloidal Gold as Labels in Lateral Flow Immunochromatographic Assays for the Detection of T-2 Toxin

**DOI:** 10.3390/molecules21010027

**Published:** 2015-12-28

**Authors:** Xiya Zhang, Chao Wu, Kai Wen, Haiyang Jiang, Jianzhong Shen, Suxia Zhang, Zhanhui Wang

**Affiliations:** 1 Beijing Advanced Innovation Center for Food Nutrition and Human Health, College of Veterinary Medicine, China Agricultural University, Beijing 100193, China; zhangxiya@cau.edu.cn (X.Z.); xyzshuishou-899@163.com (C.W.); wenkai@cau.edu.cn (K.W.); haiyang@cau.edu.cn (H.J.); sjz@cau.edu.cn (J.S.); suxia@cau.edu.cn (S.Z.); 2 Beijing Key Laboratory of Detection Technology for Animal-Derived Food Safety, College of Veterinary Medicine, China Agricultural University, Beijing 100193, China; 3 Beijing Laboratory for Food Quality and Safety, College of Veterinary Medicine, China Agricultural University, Beijing 100193, China; 4 National Reference Laboratory for Veterinary Drug Residues, College of Veterinary Medicine, China Agricultural University, Beijing 100193, China

**Keywords:** monoclonal antibody, colloidal gold, fluorescent microsphere, lateral-flow immuno-chromatographic assay, T-2 toxin

## Abstract

A new highly specific and sensitive monoclonal antibody (MAb) to T-2 toxin (T-2) was produced, providing an IC_50_ value of 1.02 ng/mL and negligible cross-reactivity (CR) to other related mycotoxins. Based on the new MAb, a lateral-flow immunochromatographic assay (LFIA) using colloidal gold (CG) and fluorescent microspheres (FMs) as labels was proposed for T-2. Under the optimized conditions, in rapid qualitative assay, the cut-off values of the CG-LFIA were 400 μg/kg in rice and 50 μg/L in fresh milk, and the cut-off values of the FMs-LFIA were 100 μg/kg in both rice and chicken feed. For the quantitative assay with the FMs-LFIA, the limit of detection (LOD) were 0.23 μg/kg and 0.41 μg/kg in rice and chicken feed, respectively, and the average recoveries ranged from 80.2% to 100.8% with the coefficient of variation (CV) below 10.8%. In addition, we found that the CG-LFIA could tolerate the matrix effect of fresh milk better than the FMs-LFIA, while the FMs-LFIA could tolerate the matrix effect of chicken feed better than CG-LFIA under the same experimental conditions. These results provide a certain reference for the selection of appropriate labels to establish a rapid LFIA in various biological samples.

## 1. Introduction

T-2 toxin (T-2), a type A trichothecene, is mainly produced by *Fusarium* species [1]. It is a ubiquitous contaminant of cereals and processed foods, occurring mainly in cold climate regions or during wet storage conditions [2,3,4]. Acute T-2 poisoning causes nausea, dizziness, vomiting, chills, abdominal distension, abdominal pain, thoracic stuffiness, diarrhea and shock-like syndrome [5,6]. Furthermore, T-2 is associated with deoxyribonucleic acid (DNA) damage [7], induction of apoptosis [8] and inhibition of protein synthesis [9]. Several studies have shown that T-2 and even some of its metabolites were toxic [10,11,12]. Once exposed to it, no effective solution is available to avoid the hazard [13], thus a rapid, sensitive and accurate analytical method must be necessarily established.

Several analytical methods for detecting T-2 have been reported, including high-performance liquid chromatography (HPLC) with a fluorescence detector and liquid chromatography-tandem mass spectrometry (LC-MS/MS) [14,15,16]. However, those methods are unsuitable for high-throughput screening of large numbers of samples because they are time consuming and labor intensive. Some enzyme-linked immunosorbent assay (ELISA) methods have also been reported for T-2 screening [13,17,18]. ELISA method still require labor-intensive operations, including incubation, washing and enzymatic reactions [19]. Recently, lateral-flow immunochromatographic assays (LFIA) are becoming increasingly popular as an efficient screening method for conducting onsite tests because of their simplicity, speed, specificity and sensitivity [20]. Compared with ELISA, the LFIA results can be obtained within 3–10 min. However, limited literatures on LFIA methods to detect T-2 residues could be found [21,22,23]. A highly specific anti-T-2 monoclonal antibody (MAb) which could distinguish T-2 and HT-2 has been produced [13,23], but the IC_50_ value of the anti-T-2 MAb was 23 ng/mL, lacking enough sensitivity for the development of LFIA. To establish a better LFIA for screening T-2, the preparation of a higher specificity and sensitivity MAb is necessary.

Colloidal gold have been commonly used as labels in LFIA (CG-LFIA) in the field of food safety [19,20,24]. But CG was only suitable for high concentrations of analyte due to the low assay sensitivity. Recently fluorescent microspheres (FMs) were reported as attractive labels in LFIA (FMs-LFIA) for their stable configuration and high fluorescence intensity [25]. The practical advantages of FMs could enhance the sensitivity of the LFIA [26], however, no comparative evaluations of FMs-LFIA *vs.* CG-LFIA for matrix tolerance in different biological samples has been conducted.

In present work, an anti-T-2 MAb with high specificity and sensitivity was produced and LFIA- labelled CG and FMs were developed to detect T-2. Moreover, to fully determine the feasibility of the proposed assay, the tolerance of the two labels for different matrices was estimated.

## 2. Results and Discussion

### 2.1. MAb Production

**Figure 1:**
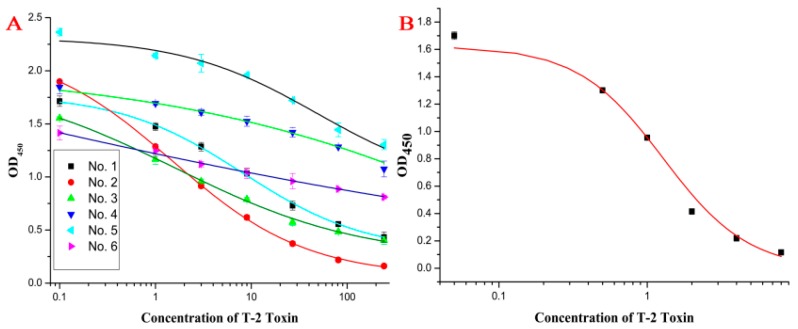
Standard curves. (**A**) represents the results of the immunization from the six BALB/c mice; and (**B**) represents the standard curve of T-2 in PBS.

The mice antisera were collected 7 days after the third immunization and characterized by an indirect competitive ELISA (icELISA). The inhibition curves of the six antisera are shown in Figure 1A. Mouse No. 2 was sacrificed for fusion because of its highest affinity to T-2. The hybridoma with the highest inhibition to T-2 was cloned three times by the limiting dilution method and further expanded for characterization. A standard curve of the best Mab, named 9C7, with an IC_50_ value of 1.02 ng/mL is shown in Figure 1B. The cross-reactivity (CR) of the MAb towards HT-2, T-2 triol, T-2 tetraol, NEO, DON and NIV were definitely lower than 0.1%. Like the MAb reported previously [13], MAb 9C7 was highly specific to T-2, while the sensitivity of MAb 9C7 was approximately 20 times higher. The selection of KLH as the carrier protein could induce high affinity [27], accounting for this phenomenon.

### 2.2. LFIA Optimization

**Figure 2:**
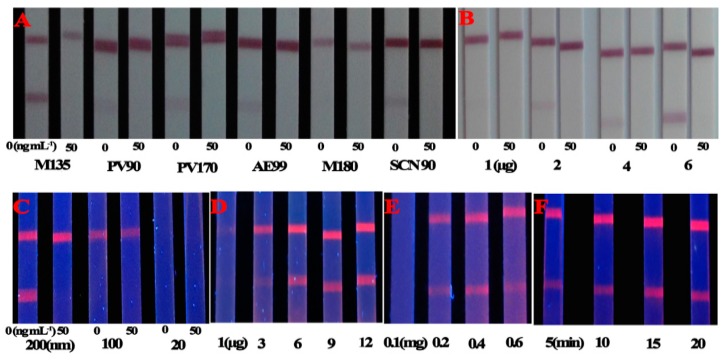
LFIA optimization. (**A**) Different NC membranes; (**B**) Amount of MAb for conjugation with CG; (**C**) FMs diameter; (**D**) Amount MAb loaded onto FMs; (**E**) Amounts of EDC; (**F**) Incubation time.

To optimize the sensitivity and clear red color of the CG-LFIA, the pH of the gold nanoparticles solution, the amounts of the MAb and the coating antigen and several NC membranes from different manufacturers were evaluated as described previously [16]. pH 8.5 of the gold nanoparticles solution, the M135 NC membrane (Figure 2A), 6 μg of MAb (Figure 2B) and 1 mg/mL of the T-2-OVA were selected as the optimized conditions for highest color intensity and the inhibition to T-2.

Given the effect of FMs-anti-T-2-MAb on assay sensitivity, the following three parameters were optimized as described previously [25,28,29,30]. Two hundred nm-diameter FMs were selected for their strong signals and inhibition of T-2 (Figure 2C). When the amount of MAb loaded on the FMs was lower than 6 μg, the fluorescence intensity and the amount of MAb were positively correlated (Figure 2D). An amount of 0.4 mg of the EDC was suitable for the conjugation of FMs and MAb (Figure 2E). Negative or positive results could be obtained immediately under the UV-Light, and the quantitative analysis was realized by an ESE-Quant LFR fluorescence reader. The fluorescence intensity increased rapidly during the first 15 min and then remained stable at 15–20 min. Thus, LFIA assays should be dried for 15 min at 37 °C before analysis (Figure 2F), which was consistent with the data of reference [30].

**Figure 3:**
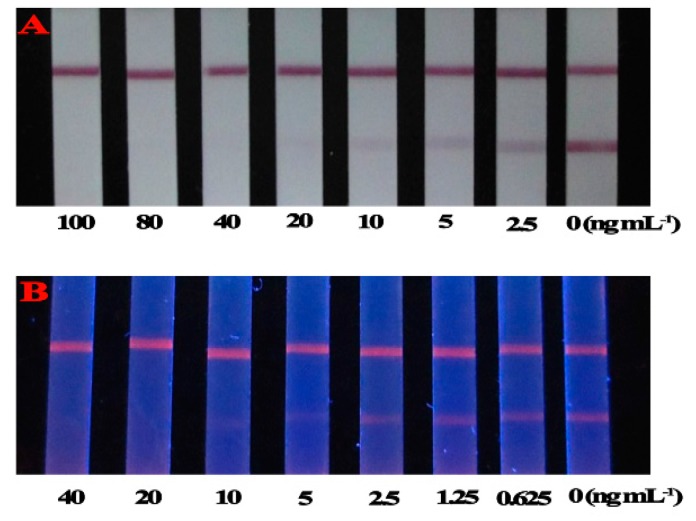
Detection of T-2 in PBS by the CG-LFIA (**A**); and FMs-LFIA (**B**).

**Figure 4:**
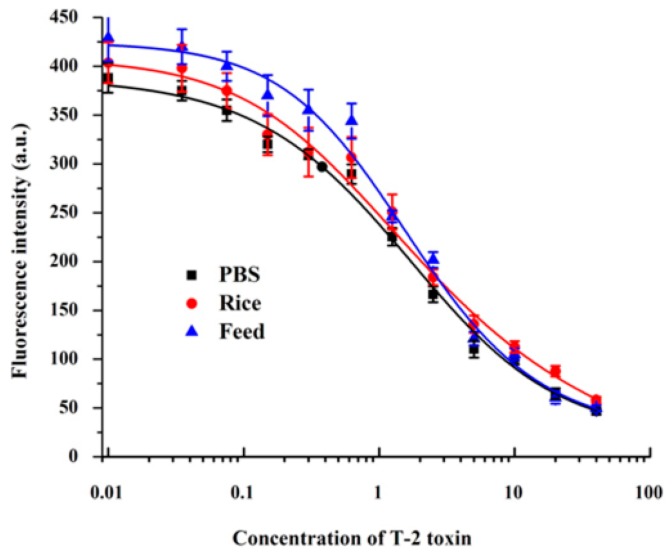
Calibration curves for T-2 in PBS, rice and chicken feed by the FMs-LFIA.

Based on the optimized conditions, the cut-off values of the CG-LFIA and FMs-LFIA in 0.01 M PBS (pH 7.4) to the naked eye were 40 ng/mL and 10 ng/mL, respectively (Figure 3). The sensitivity of the FMs-LFIA was four times higher than that of the CG-LFIA, which agreed with the literature [21,24]. The IC_50_ value of T-2 in PBS by FMs-LFIA, presented in Figure 4 and Table 1, meets the requirements of residue detection.

**Table 1:** LOD, IC_50_, IC_20_–IC_80_ and R^2^ of T-2 in PBS, rice and chicken feed by FMs-LFIA.

	PBS	Rice	Chicken Feed
LOD	0.28	0.23	0.41
IC_50_ (ng/mL)	1.58	1.78	1.78
IC_20_~IC_80_ (ng/mL)	0.28–8.9	0.23–13.7	0.41–7.8
R^2^	0.990	0.987	0.989

### 2.3. Comparison between CG-LFIA and FMs-LFIA for Matrix Effect

**Figure 5:**
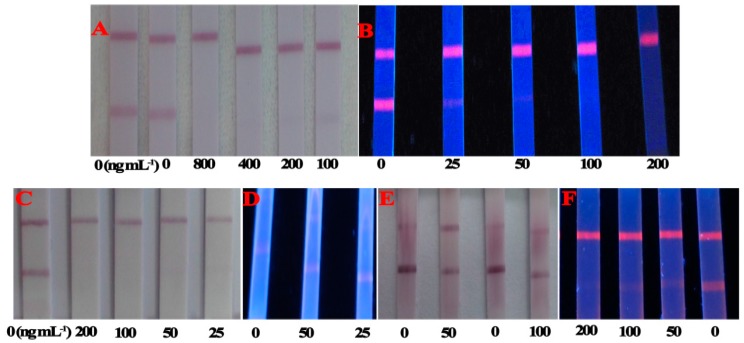
Detection of T-2 in rice (**A**); fresh milk (**C**); and chicken feed (**E**) by the CG-LFIA; and in rice (**B**); fresh milk (**D**); and chicken feed (**F**) by the FMs-LFIA, respectively.

To compare the matrix effect of CG and FMs as labels in LFIA, T-2 was spiked in rice, fresh milk and chicken feed. As shown in Figure 5A,B, the cut-off values of the CG-LFIA and FMs-LFIA to the naked eye in rice were 400 μg/kg and 100 μg/kg, respectively. The cut-off value of the CG-LFIA was higher than the reported value, whereas for FMs-LFIA it was almost the same as in [22,23]. The cut-off value of the CG-LFIA was 50 μg/L in fresh milk (Figure 5C). In comparison, the FMs-LFIA result was significantly influenced by the milk matrix (Figure 5D), which might due to a high level of sugar and protein in fresh milk. A better result could be acquired if the fresh milk were diluted five times with PBS (containing 0.05% Tween 20) [25,29], whereas the decreasing sensitivity, followed like a shadow. The matrix effect in chicken feed for LFIAs was the opposite: the cut-off value of the FMs-LFIA was 100 μg/kg (Figure 5F), but CG-LFIA could not tolerate the matrix (Figure 5E). These results indicated that both LFIAs could tolerate the rice matrix, but the CG could tolerate milk matrix better than FMs, whereas the FMs could tolerate chicken feed matrix better than CG under the same experimental conditions. The exact reasons for these results were still unknown.

A quantitative method to detect T-2 was established in rice and chicken feed with the FMs-LFIA. The matrix standard curves were nearly indistinguishable from the standard curve constructed in PBS (Figure 4), indicating that the extraction method was reasonable. The parameters of the standard curves in PBS, rice and chicken feed are shown in Table 1. In PBS, rice and chicken feed, the IC_50_ values were 1.58 ng/mL, 1.78 ng/mL and 1.78 ng/mL, respectively, while the corresponding limits of detection (LODs) were 0.28 μg/kg, 0.23 μg/kg and 0.41 μg/kg. In present study, the LODs in rice and chicken feed was similar to, or even lower than those of published methods [21,22,23]. Recoveries, listed in Table 2, ranged from 80.2% to 100.8%, with coefficients of variation (CV %) under 10.8% in rice and chicken feed.

**Table 2:** Recoveries and CV values for T-2 in rice and chicken feed.

Sample	Spiked (μg/kg)	Test (μg/kg)	Recoveries (%)	CV (%)
Rice	5	4.748	94.9	5.42
	10	10.08	100.8	7.51
	20	19.22	96.1	9.78
Chicken Feed	5	4.02	80.2	10.8
	10	8.901	89	9.87
	20	17.56	87.8	7.56

## 3. Experimental Section

### 3.1. General Information

T-2 toxin, HT-2 toxin, deoxynivalenol (DON), neosolaniol (NEO), nivalenol (NIV), T-2-triol, T-2-tetraol, keyhole limpet hemocyanin (KLH), bovine serum albumin (BSA), ovalbumin (OVA), Freund’s incomplete adjuvant (FIA), Freund’s complete adjuvant (FCA), PEG1500, hypoxanthine aminopterin thymidine (HAT), and a mouse MAb isotyping kit were obtained from Sigma-Aldrich (St. Louis, MO, USA). Peroxidase-conjugated goat antimouse IgG was obtained from Jackson ImmunoResearch Laboratories, Inc. (West Grove, PA, USA). Nitrocellulose membranes (Millipore 135 and Millipore 180) were purchased from Millipore (Bedford, MA, USA). Nitrocellulose membrane (Pall Vivid PV90 (PV90), Pall Vivid PV170 (PV170), Sartorius CN 90 (SCN90) and Whatman AE99 (AE99)) were purchased from Shanghai JieYi Biological Technology Co. Ltd. (Shanghai, China). FluoSpheres^®^ Carboxylate-Modified Microspheres (200 nm, 100 nm, 20 nm, red fluorescent (580/605 nm, Ex/Em), 2% solids) were obtained from Invitrogen (Carlsbad, CA, USA). The sample pad (CFKJ-0328) and the absorbance pad (CH37K) were supplied by Shanghai Liangxin Co. Ltd. (Shanghai, China). 1-[3-(Dimethylamino) propyl]-3 ethylcarbodiimide hydrochloride (EDC∙HCl), and *N*-hydroxysuccinimide (NHS) were purchased from Aladdin Chemistry Co. Ltd. (Shanghai, China). The other reagents and solvents were of analytical grade or higher. Eight-week-old female BALB/c mice were obtained from Vital River Laboratory Animal Technology Co. Ltd. (Beijing, China) and raised under strictly controlled conditions. The experimental procedures involving animals in this study were approved by the Animal Care Center of the China Agricultural University, Beijing, China.

### 3.2. Preparation of Anti-T-2 MAb

The T-2 toxin was coupled with the carrier proteins KLH or OVA by using the active ester method as described previously [13,31,32]. Six female 8-week-old BALB/c mice were injected subcutaneously three times with the immunogen of T-2-KLH [24]. The mouse that produced a high titer of antibodies and showed competitive inhibition with T-2 was sacrificed for fusion. Spleen cells from the immunized mouse were fused with Sp2/0 cell using PEG1500 [31,33,34]. The supernatants of the hybridoma were collected and screened by icELISA described below. The positive hybridomas were subcloned three times by limiting dilution method. Ascites fluids were produced and purified by the ammonium sulphate precipitation.

### 3.3. Indirect Competitive ELISA (icELISA)

Microplates were coated with 100 μL/well of T-2-OVA (50 ng/well) in 0.05 M carbonate bicarbonate buffer (CB, pH 9.6) and then incubated at 4 °C for 8 h. The plates were blocked with 200 μL of 1% BSA in PBS (blocking buffer) and incubated at 37 °C for 2 h. After the blocking buffer was discarded, 50 μL/well of T-2 standard was serially diluted in PBS at 0, 0.5, 1, 2, 4 and 8 ng/mL and 50 μL/well of MAb (40,000-fold diluted in PBS) was added and incubated for 30 min at 37 °C. Other steps were described as the previous reference [29,35]. In order to evaluate the specificity of the MAb, the inhibitions of binding of the MAb with HT-2, T-2-triol, T-2-tetraol, DON, NEO and NIV were tested. The cross-reactivity (CR) values were calculated as follows:

(1) CR = (IC_50_ of T-2/IC_50_ of competitor) × 100%

### 3.4. Preparation of CG-Anti-T-2-MAb Conjugates

Uniform gold nanoparticles (40 nm in diameter) were synthesized as described previously [16,17]. K_2_CO_3_ (0.1 mol/L) was used to adjust the pH of the gold nanoparticles solution to 8.5 for conjugation with MAb. Six μg of the MAb was added dropwise to 1 mL of pH-adjusted gold nanoparticles solution with gentle stirring. The mixture was reacted for 10 min and blocked by 20 μL 20% (*w*/*v*) filtered BSA for another 10 min. Then the mixture was centrifuged at 8000 *g* for 10 min at 4 °C and the pellets were re-suspended by adding 1 mL of 0.01 M PBS (pH 7.4) with 0.5% BSA, 0.2% PVP, 2% sucrose and 0.5% Tween-20.

### 3.5. Preparation of FMs-Anti-T-2-MAb Conjugates

The anti-T-2 MAb was conjugated to FMs according to the carboxylate-modified microspheres method [20,23,24,30]. Briefly, 20 μL of 2% FMs was suspended in 1 mL of 0.05 M MES (pH 6.5) with 0.4 mg of EDC and 6 μg of anti-T-2 MAb under dark conditions for 2 h at 25 °C. Then 200 μL of 0.1 M glycine was added and incubated for another 30 min in order to terminate the reaction, and the mixtures were centrifuged at 8000 *g* for 15 min at 4 °C. The precipitates were resuspended in 200 μL of 0.05 M PBS containing 1% BSA, 2% PEG 20,000 and 1% Dextran 4000. Finally the suspensions were stored at 4 °C in the dark and sonicated for 5 min before use.

### 3.6. Assembly of the LFIA Components and Test Procedure

The components of the test strip consisted of three sections, including NC membrane coated with the T-2-OVA (1 mg/mL) and the goat anti-mouse antibody (8.1 mg/mL), absorbent pad and sample pad. The assembly procedure was similar as described previously [16,19,20]. Finally, the whole assembled plate was cut into 3 mm width strips and stored under dry conditions at room temperature.

The principle of LFIA was based on the competitive binding of T-2 and T-2-OVA to the MAb labelled with CG or FMs. Briefly, 2 μL of CG-MAb conjugates or FMs-MAb conjugates and 200 μL of the standard solution or samples for CG-LFIA (or 120 μL for FMs-LFIA) were added into one well of the 96-microtiter plate and mixed for 3 min. Then the strip was vertically inserted into the corresponding micro-well for another 10 min for CG-LFIA (or FMs-LFIA). The C-line was colored to ensure that the procedures of the LFIA were correct. For the CG-LFIA, the result was obtained immediately. For the FMs-LFIA, the result was obtained directly at UV-Light whose excitation wavelength was set at 365 nm, or the test strips should be dried at 37 °C for another 15 min before testing by using the ESE-Quant reader whose excitation wavelength was at 580 nm and emission wavelength was at 605 nm.

### 3.7. Assay of T-2 in Rice, Chicken Feed and Fresh Milk by LFIA

Rice or chicken feed (1 g) was weighed into 10 mL polypropylene centrifuge tubes. Methanol (20% *v*/*v*, 2 mL for rice or 3 mL for chicken feed) was added for extraction. The mixture was vortexed for 3 min and centrifuged at 3000 *g* for 10 min. Then the supernatant was diluted two times by 0.01 M PBS (pH 7.4) for the detection of GC-LFIA and FMs-LFIA. T-2-free fresh milk samples were supplied by the National Reference Laboratory for Veterinary Drug Residues (Beijing, China) and those samples were directly used for analysis by the GC-LFIA and FMs-LFIA methods without further extraction steps.

## 4. Conclusions

In summary, we have developed a rapid LFIA using CG and FMs as labels based on a new MAb for the detection of T-2 in rice, fresh milk and chicken feed. In addition, from the examination of how the two labels tolerate different biological matrix effects, we found that both labels in LFIA could tolerate the rice matrix, but the CG could tolerate the milk matrix better than FMs, whereas the FMs could tolerate the chicken feed matrix better than CG. These results provide a reference for the selection of appropriate labels to establish a rapid LFIA in different biological samples.
